# Retraction: *Mycobacterium indicus pranii (Mw)* Re-Establishes Host Protective Immune Response in *Leishmania donovani* Infected Macrophages: Critical Role of IL-12

**DOI:** 10.1371/journal.pone.0336086

**Published:** 2025-11-05

**Authors:** The PLOS One Editors

After this article [1] was published, concerns were raised regarding results presented in Fig 4.

Specifically:

- In the Fig 4A UIM p-ERK1/2 panel, lanes 3 and 4 appear similar
- The Fig 4A UIM TotalERK1/2 panel appears similar to the Fig 4B UIM TotalERK1/2 panel despite representing different results
- There appear to be one or more vertical discontinuities in the following panels:
  - Fig 4B IM p-p38 MAPK
  - Fig 4B IM TotalERK1/2

Author BS disagreed with the above concerns and stated that the vertical discontinuities in Fig 4B could be caused by technical artifacts. They stated the data underlying [1] are no longer available.

In light of the above concerns that question the reliability of the results and which cannot be resolved in the absence of the underlying data, the *PLOS One* Editors retract this article.

BS did not agree with the retraction and stands by the article’s findings. AA, GG, SaikatM, SBanerjee, SBhattacharjee, PB, SK, SH, SBM, and SubrataM either did not respond directly or could not be reached.
